# USP22 Promotes NSCLC Tumorigenesis via MDMX Up-Regulation and Subsequent p53 Inhibition

**DOI:** 10.3390/ijms16010307

**Published:** 2014-12-25

**Authors:** Fangbao Ding, Chunrong Bao, Yue Tian, Haibo Xiao, Mingsong Wang, Xiao Xie, Fengqing Hu, Ju Mei

**Affiliations:** 1 Department of Cardiothoracic Surgery, Xinhua Hospital, School of Medicine, Shanghai Jiaotong University, Shanghai 200092, China; E-Mails: bcrcar@126.com (C.B.); xbcardio@163.com (H.X.); wmso126@126.com (M.W.); xixe126@126.com (X.X.); hqng126@126.com (F.H.); mju126@126.com (J.M.); 2 Institute of Orthopaedics, Chinese PLA General Hospital, Beijing 100853, China; E-Mail: tyuecar@126.com

**Keywords:** USP22, MDMX, p53, NSCLC

## Abstract

Increasing evidence suggests that ubiquitin-specific protease 22 (USP22) has great clinicopathologic significance in oncology. In this study, we investigated the role of USP22 in human NSCLC tumorigenesis along with the underlying mechanisms of action. First, we determined the expression of USP22 in human NSCLC, as well as normal tissues and cell lines. We then studied the effects of USP22 silencing by shRNA on NSCLC cell growth *in vitro* and tumorigenesis *in vivo*, along with the effect on the p53 pathway. We found that USP22 is overexpressed in human NSCLC tissues and cell lines. USP22 silencing by shRNA inhibits proliferation, induces apoptosis and arrests cells at the G0/G1 phases in NSCLC cells and curbs human NSCLC tumor growth in a mouse xenograft model. Additionally, USP22 silencing downregulates MDMX protein expression and activates the p53 pathway. Our co-immunoprecipitation analysis shows that USP22 interacts with MDMX in NSCLC cells. Furthermore, MDMX silencing leads to growth arrest and apoptosis in NSCLC cells, and over-expression of MDMX reverses the USP22 silencing-induced effects. Taken together, our results suggest that USP22 promotes NSCLC tumorigenesis *in vitro* and *in vivo* through MDMX upregulation and subsequent p53 inhibition. USP22 may represent a novel target for NSCLC treatment.

## 1. Introduction

Worldwide, lung cancer is the most frequently diagnosed cancer (12.7%) and the most common cause of cancer mortality (18.2%), accounting for 1.38 million deaths in 2008 [1]. Lung cancer is a very aggressive malignancy associated with high mortality and low cure rates [1,2]. Non-small cell lung cancer (NSCLC), which mainly includes adenocarcinoma, squamous cell carcinoma and large-cell lung carcinoma, accounts for approximately 80%–85% of all lung cancers [3]. As a class, NSCLCs are usually not very sensitive to chemotherapy and/or radiation; thus, surgery with adjuvant chemotherapy is the treatment of choice if diagnosed at an early stage. However, there is a high risk of relapse after the initial surgery, and adjuvant chemotherapy is associated with a survival advantage of only about 5% at five years [4]. In recent years, with the growing insight into the pathophysiology of NSCLC, tremendous efforts have been made to identify and develop new agents to improve disease management. A few novel drugs targeting EGFR, VEGF-A and ALK have been approved, but some other agents failed in clinical trials [5,6]. While the value of these newly-approved drugs remains to be fully elucidated, it is imperative to identify new pathways and therapeutic targets that can provide alternative effective agents to improve the prognosis of NSCLC.

Ubiquitin-specific protease 22 (USP22) is one of >50 ubiquitin-specific proteases (USPs) that remove ubiquitin moieties from target proteins [7]. USPs stabilize substrate proteins by inhibiting their ubiquitin-dependent degradation. USP22 was initially identified in 2005 as a key component of the “death-from-cancer” signature, an 11-gene signature predicting for recurrence, metastasis and therapy resistance in multiple cancers [8]. Later studies demonstrate that USP22 is a subunit of the human SAGA (Spt-Ada-Gcn5 acetyltransferase) transcriptional cofactor complex and activates gene transcription for cell-cycle progression through deubiquitylation of histones H2A and H2B [9,10]. USP22 has also been shown to promote cell proliferation through activating oncogenic proteins, such as BMI-1, c-MYC and Sirt1 [9,11,12], and antagonizing tumor suppressors, such as p53 [12,13]. An increasing body of literature shows that USP22 expression is elevated in cancers and is prognostic for disease progression and treatment outcome [14,15,16,17,18,19,20]. Particularly, results from two research groups in 2012 demonstrate that the USP22 level is increased and associated with overall survival in NSCLC patients [15], as well as patients with early-stage NSCLC [17], implying the involvement of USP22 in this specific type of cancer. However, whether USP22 promotes tumorigenesis in NSCLC remains unclear. In the present study, we compared the expression of UPS22 in human NSCLC *vs.* normal tissues and cell lines. We then studied the effects of UPS22 silencing on NSCLC cell growth, cell cycle distribution and apoptosis *in vitro* and xenograft tumor growth *in vivo*. Furthermore, we elucidated the role of MDMX and p53 in USP22’s regulatory effect in NSCLC.

## 2. Results

### 2.1. Expression Patterns of USP22, MDMX and p53 in NSCLC

**Figure 1:**
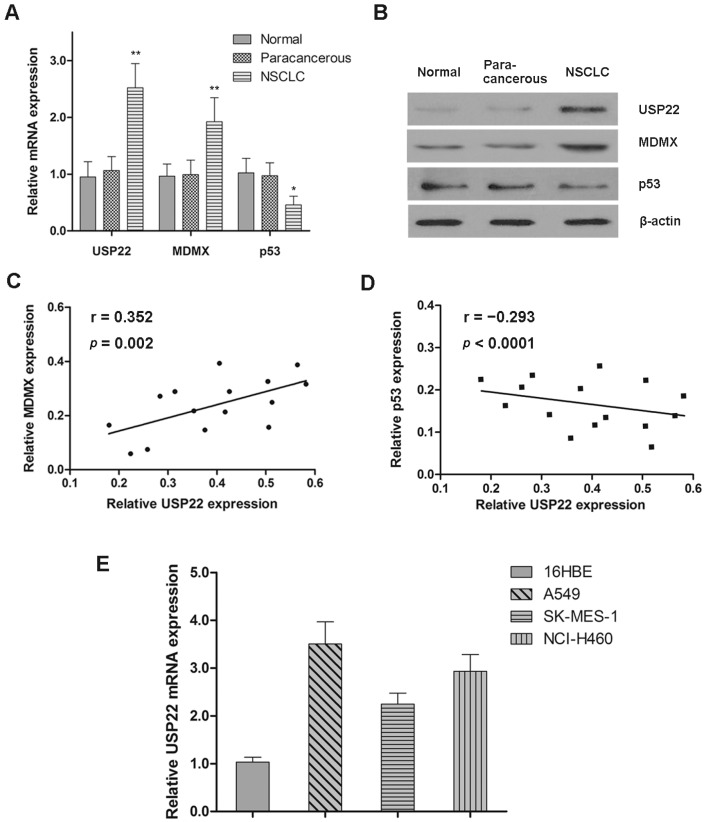
Expression patterns of USP22, MDMX and p53 in NSCLC. (**A**,**B**) The mRNA and protein expressions of USP22, MDMX and p53 in normal, paracancerous and NSCLC tissues (*n =* 15); (**C**,**D**) Pearson correlation analysis of the protein expressions between USP22 and MDMX/p53 in NSCLC tissues; (**E**) the relative mRNA expression of USP22 in 16HBE and NSCLC cell lines (A549, SK-MES-1 and NCl-H460) normalized to β-actin (*n =* 3). Data are expressed as the means ± SD. * *p* < 0.05, ** *p* < 0.01.

The mRNA and protein expressions of USP22, MDMX and p53 in normal, paracancerous and NSCLC tissues were determined by qRT-PCR and western blot analysis, respectively. We found no differences in UPS22, MDMX and p53 mRNA and protein levels between normal and paracancerous tissues; however, UPS22 and MDMX levels were significantly higher, while the p53 level was lower in NSCLC tissues (Figure 1A,B). Moreover, there was a positive correlation between the protein levels of USP22 and MDMX in NSCLC tissues (*r =* 0.352, *p* = 0.002), while a negative correlation between that of USP22 and p53 (*r =* −0.293, *p* < 0.0001). We also found significantly higher mRNA levels of USP22 in the three NSCLC cell lines (A549, SK-MES-1 and NCl-H460) than the noncancerous human bronchial epithelial cell line (16HBE) (Figure 1E). Therefore, UPS22 is overexpressed in human NSCLC tissues and cell lines, both *in vivo* and *in vitro*.

### 2.2. USP22 Silencing Inhibits Proliferation and Induces Apoptosis and Cell Cycle Arrest in NSCLC Cells

**Figure 2:**
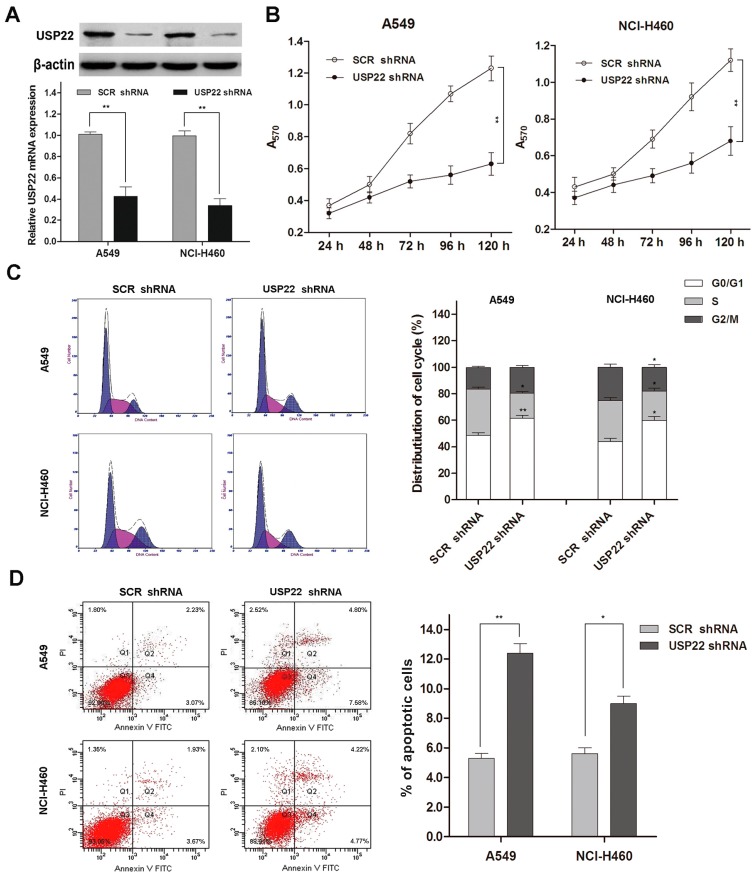
USP22 silencing suppresses proliferation and induces apoptosis and cell cycle arrest in NSCLC cells. (**A**) USP22 mRNA and protein expressions in A549 and NCl-H460 cells transfected with USP22 shRNA or scrambled control shRNA (SCR shRNA) (*n =* 3); (**B**) cell proliferation in transfected A549 and NCl-H460 cells by the MTT assay (*n =* 3); (**C**) cell cycle distribution by flow cytometry with PI staining (*n =* 3). Histograms are representative of three independent experiments; (**D**) Cell apoptosis by flow cytometry with Annexin V-FITC and PI double staining (*n =* 3). The values of the Q2 plus Q4 quadrant represent apoptosis rates. Data are expressed as the means ± SD. * *p* < 0.05 *vs.* SCR shRNA, ** *p* < 0.01 *vs.* SCR shRNA.

We studied the impact of UPS22 silencing on A549 and NCI-H460 cell growth and apoptosis using shRNA transfection. USP22 shRNA transfection down-regulated both the mRNA and protein expressions of USP22 in A549 and NCI-H460 (Figure 2A). We then studied cell proliferation using the MTT assay and cell cycle distribution and apoptosis using flow cytometry. Our data showed that USP22 silencing led to significantly slower cell growth compared with the control (*p* < 0.01 after 120 h) (Figure 2B). Meanwhile, our flow cytometry analysis revealed that, compared with the control, USP22 shRNA-transfected cells displayed a significantly higher portion of cells at the G0/G1 phases and significantly lower portions of cells at the S and G2/M phases (Figure 2C), indicating that USP22 silencing induces cell cycle arrest. Additionally, cells transfected with USP22 shRNA showed significantly increased apoptosis compared with the control (Figure 2D).

### 2.3. USP22 Silencing Down-Regulates MDMX and Up-Regulates the p53 Pathway in NSCLC Cells

**Figure 3:**
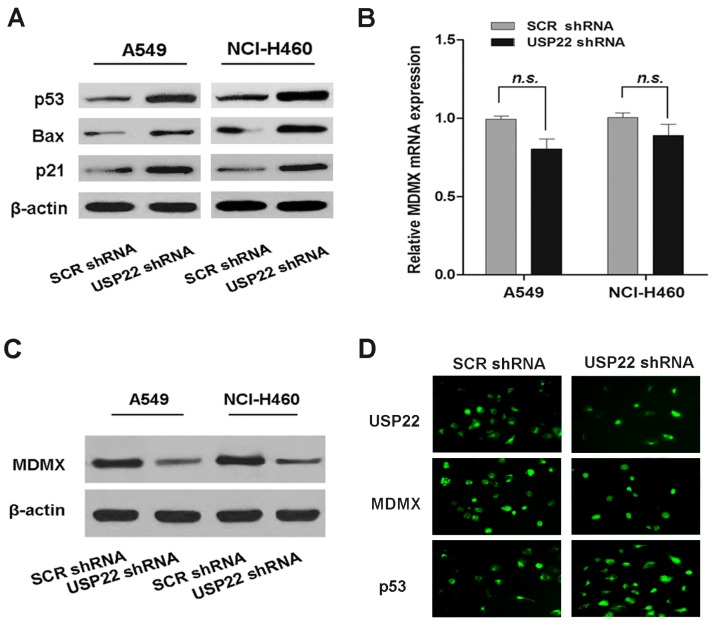
USP22 silencing activates the p53 pathway, down-regulates MDMX protein expression and interacts with MDMX in NSCLC cells. (**A**) The levels of p53 pathway proteins in A549 and NCl-H460 cells transfected with USP22 shRNA or SCR shRNA; (**B**,**C**) the mRNA and protein expressions of MDMX in A549 and NCl-H460 cells transfected with USP22 shRNA or SCR shRNA (*n =* 3). β-actin was used as an internal control. Data are expressed as the means ± SD. n.s. indicates no significant difference; (**D**) Immunofluorescence (IF) analysis of USP22, MDMX and p53 expression in A549 cells transfected with USP22 shRNA or SCR shRNA.

Having demonstrated that USP22 regulates cell proliferation in NSCLC cells, we investigated the possible underlying mechanisms. Abnormalities in p53 are frequently found in lung cancer, indicating its importance in this malignancy [21]. We noted that USP22 has been reported to antagonize the p53 pathway in previous studies [12,13]. To find out whether p53 is involved in the regulatory function of USP22 in NSCLC, we assessed p53 activation in USP22 shRNA-transfected cells by Western blot analysis. We found that USP22 silencing in A549 and NCI-H460 cells increased the protein expression of p53, p21 and Bax, the key p53 signal molecules (Figure 3A), suggesting that p53 activation plays a role in USP22 silencing-induced growth inhibition. MDM2 and MDMX promote ubiquitin-dependent p53 degradation and are the two major negative regulators of p53. It has been reported that USP22 antagonizes p53 in bladder cancer cells through up-regulating MDM2 [13]. Interestingly, we found that in A549 and NCI-H460 cells, USP22 silencing, while activating the p53 pathway, decreased MDMX protein expression (Figure 3C), which was confirmed with immunofluorescence (IF) analysis (Figure 3D). However, the mRNA expression of MDMX was not affected (Figure 3B). On the bases of these results, we speculated that USP22 silencing activates the p53 pathway in NSCLC cells by post-transcriptional down-regulation of MDMX.

### 2.4. MDMX Directly Binds to USP22 in NSCLC Cells

**Figure 4:**
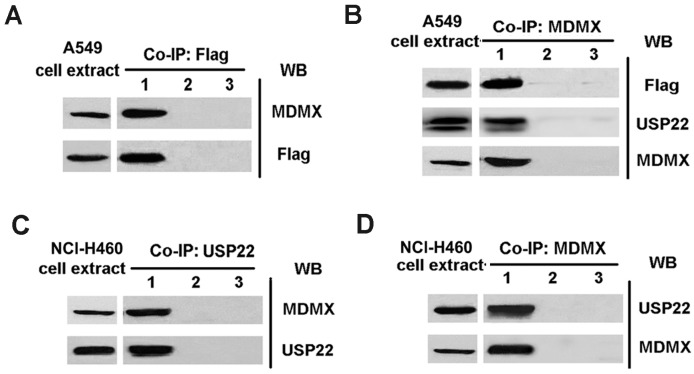
USP22 interacts with MDMX in NSCLC cells. (**A**) The cell lysates from A549-USP22 were immunoprecipitated with anti-Flag antibody and immunoblotted with anti-Flag and anti-MDMX antibodies, respectively. Lane 1, Flag-immunoprecipitates; Lane 2, isotype control antibody-immunoprecipitates; Lane 3, PBS-immunoprecipitates; (**B**) The cell lysates from A549-USP22 cells were immunoprecipitated with anti-MDMX antibody and immunoblotted with anti-MDMX, anti-Flag and anti-USP22 antibodies, respectively. Lane 1, MDMX-immunoprecipitates; Lane 2, isotype control antibody-immunoprecipitates; Lane 3, PBS-immunoprecipitates; (**C**,**D**) The cell lysates from NCl-H460 were immune-precipitated with anti-USP22 antibody (**C**) or anti-MDMX antibody (**D**) and immunoblotted with anti-Flag and anti-MDMX antibodies, respectively. Lane 1, USP22- or MDMX-immunoprecipitates; Lane 2, isotype control antibody-immunoprecipitates; Lane 3, PBS-immunoprecipitates.

To investigate the interaction between MDMX and USP22, co-immunoprecipitation analysis was performed in NSCLC cell lines. An A549 cell line stably expressing Flag-tagged USP22 (A549-USP22) was generated by retroviral infection. When A549-USP22 cell lysates were immunoprecipitated with the anti-Flag antibody and immunoblotted with anti-Flag and anti-MDMX antibodies, respectively, both Flag and MDMX were detected in the immunoprecipitates (Figure 4A). Similarly, USP22 and Flag were detected in the MDMX immunoprecipitates (Figure 4B). These data indicate that MDMX directly binds to Flag-tagged USP22 in A549-USP22 cells. Furthermore, we confirmed that MDMX directly interacts with endogenous USP22 in NCl-H460 cells, as MDMX was detected in USP22 immunoprecipitates and *vice versa* (Figure 4C,D). Our data suggest that MDMX directly binds to USP22 in NSCLC cells.

### 2.5. MDMX Mediates USP22’s Regulation Effect in NSCLC Cells

**Figure 5:**
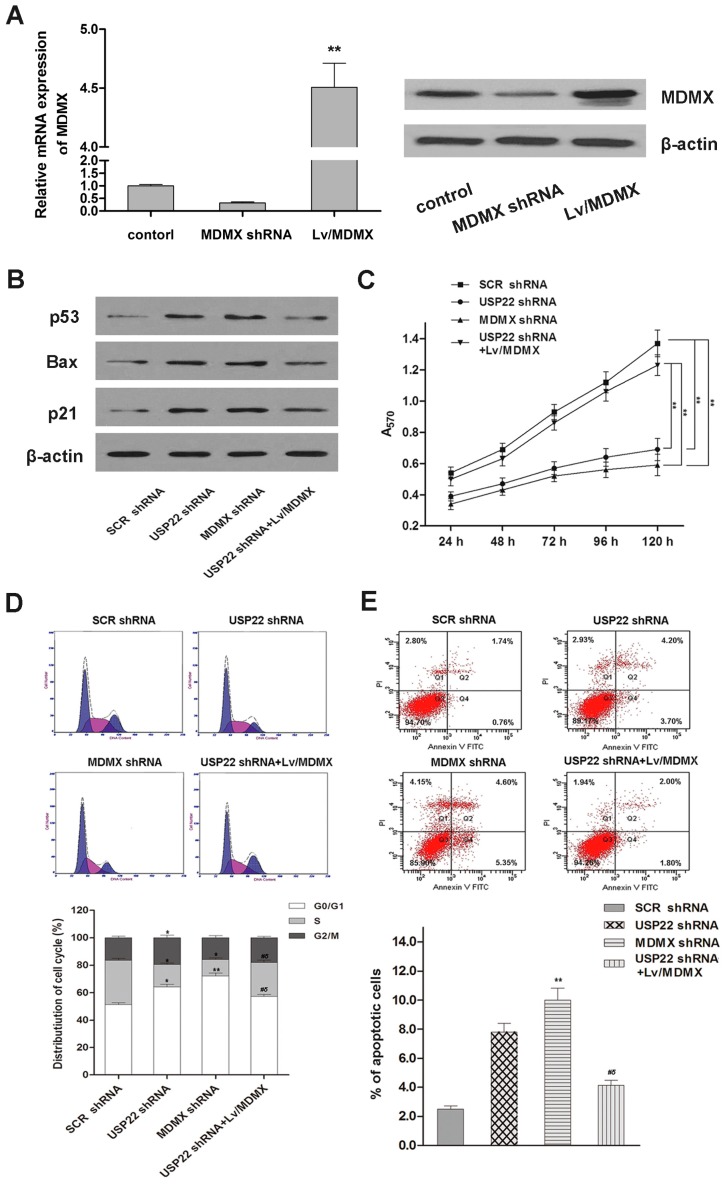
Cell proliferation, cell cycle distribution, cell apoptosis and p53 pathway in A549 cells transfected with SCR shRNA, USP22 shRNA, MDMX shRNA or co-transfected with USP22 shRNA and Lv/MDMX. (**A**) The mRNA and protein expressions of MDMX; (**B**) levels of p53 pathway proteins in A549 cells by Western blot; (**C**) cell proliferation by the MTT assay. ** *p* < 0.01; (**D**,**E**) Cell cycle distribution by flow cytometry with PI staining (**D**) and cell apoptosis by flow cytometry with Annexin V-FITC and PI double staining (**E**). * *p* < 0.05 *vs.* SCR shRNA, ** *p* < 0.01 *vs.* SCR shRNA, ^#^ *p* < 0.05 *vs.* USP22 shRNA, ^δ^ *p* < 0.05 *vs.* MDMX shRNA. Data are expressed as the means ± SD, *n =* 3.

Our findings on the relationship between USP22, p53 and MDMX in NSCLC cells suggest that MDMX may be a key mediator of USP22’s regulatory effects in NSCLC. To test this hypothesis, we studied the effects of MDMX silencing by MDMX-specific shRNA transfection in A549 cells (Figure 5A). Indeed, we found that MDMX silencing activated the p53 pathway, inhibited cell proliferation and induced apoptosis and cell cycle arrest at the G0/G1 phases, strikingly similar to that observed with USP22 silencing (Figure 5B–E). Importantly, overexpression of MDMX reversed USP22 silencing, induced p53 activation, growth inhibition, cell cycle arrest and apoptosis in A549 cells (Figure 5B–E). These results demonstrate that MDMX mediates USP22’s regulation effect in the NSCLC cell line.

### 2.6. USP22 Silencing Inhibits NSCLC Tumorigenesis in Vivo

**Figure 6:**
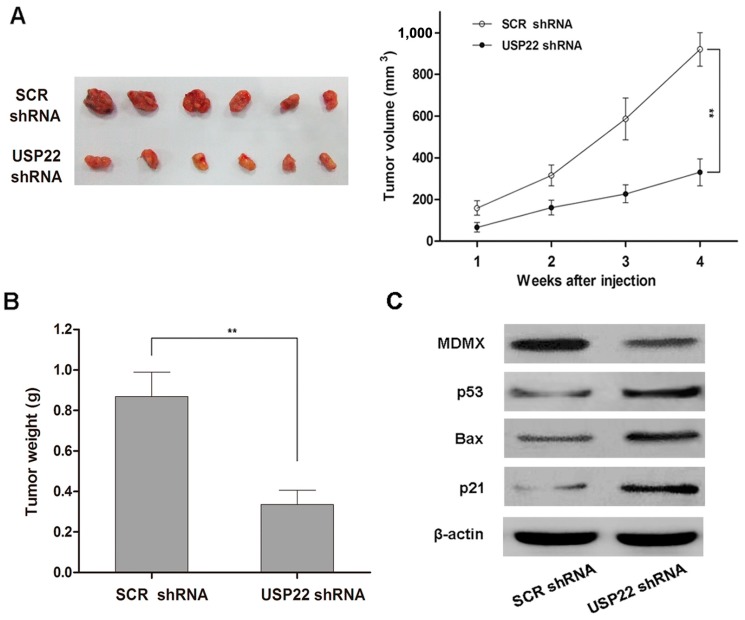
USP22 knockdown inhibits NSCLC tumorigenesis and modulates p53 pathway signals *in vivo.* (**A**) Growth of xenograft tumors derived from A549 cells transfected with USP22 shRNA or SCR shRNA in nude mice, ** *p* < 0.01; (**B**) The weight of xenograft tumors in nude mice. Data are expressed as the means ± SD, *n =* 6, ** *p* < 0.01; (**C**) Levels of MDMX and p53 pathway proteins in xenograft tumor tissues derived from A549 cells transfected with USP22 shRNA or SCR shRNA.

To investigate the relevance of our *in vitro* findings to NSCLC tumorigenesis *in vivo*, we monitored the growth of tumors derived from A549 cells transfected with USP22 shRNA or SCR shRNA in a xenograft mouse model. Consistent with our *in vitro* findings, tumors derived from USP22 shRNA-transfacted A549 cells grew at a much slower rate than those derived from SCR shRNA-transfected cells, as reflected in the significantly smaller tumor size (Figure 6A) and tumor weight (Figure 6B) four weeks after cell implantation. In addition, similar to our *in vitro* findings, USP22 silencing led to a decreased level of MDMX and increased levels of p53 pathway proteins in xenograft tumor tissues (Figure 6C). Therefore, our results from the mouse xenograft model demonstrate that USP22 silencing inhibits NSCLC tumorigenesis *in vivo* through regulating the MDMX–p53 pathway.

## 3. Discussion

USP22, a subunit of the human SAGA transcriptional complex, is required for transcriptional activation of genes controlling cell-cycle progression. Recent research advances have linked deregulation of USP22 to specific types of cancer, including NSCLC; however, the functional role of USP22 and its underlying mechanisms of action in cancer development are not well defined. In this study, we demonstrated that USP22 is overexpressed in human NSCLC tumor tissues and cell lines. Our *in vitro* and *in vivo* studies showed that USP22 silencing by shRNA inhibits proliferation and induces cell cycle arrest and apoptosis in human NSCLC cells *in vitro* and curbs human NSCLC tumor growth in a mouse xenograft model *in vivo*. Intriguingly, we found that USP22 silencing activates the p53 pathway in human NSCLC cells and tumor tissues along with downregulation of MDMX protein, a major negative regulator of p53. Our qRT-PCR and co-immunoprecipitation analysis revealed that MDMX is post-transcriptionally regulated by USP22 and directly binds to USP22 in NSCLC cells. Further experiments showed that MDMX silencing induces growth arrest, apoptosis and p53 activation in NSCLC cells, similar to that observed with USP22 silencing; and over-expression of MDMX reverses USP22 silencing-induced effects. Taken together, our results suggest that USP22 promotes NSCLC cell growth *in vitro* and NSCLC tumorigenesis *in vivo*, and these effects are through MDMX up-regulation and subsequent p53 inhibition.

The tumor suppressor p53 is a key player in cancer biology and represents an attractive target for the development of anti-cancer therapies [22]. P53 is negatively regulated by both MDM2 and MDMX. MDM2 is an E3 ubiquitin ligase that ubiquitylates p53 and leads to its proteasomal degradation. MDMX has no intrinsic ubiquitin ligase activity, but can increase MDM2 ubiquitin ligase activity by forming heteroligomers with MDM2. MDM2/MDMX heteroligomers may also bind to p53 and suppress its transcriptional activity [23]. Recent advances in biomedical research have shown that activation of p53 through targeting MDM2, MDMX or both is a viable strategy for cancer therapy [24]. Most MDM2/MDMX-targeted agents disrupt p53–MDM2 or p53–MDMX interaction or inhibit MDM2 ubiquitin ligase activity [24,25]. RG7112, a nutlin-3a derivative that inhibits p53–MDM2 interaction, is in a phase I clinical trial, and its proof-of-mechanism study in liposarcoma patients has been reported recently [26]. Several small molecule inhibitors of MDM2 ubiquitin ligase activity are currently in preclinical investigation [27,28,29]. Attempts have also been made to activate p53 via reducing MDMX protein expression. NSC207895, a small molecule inhibitor of MDMX expression, acts additively with nutlin-3a to activate p53 and induce apoptosis in MCF-7 cells [30]. 17-AAG, a small molecule HSP90 inhibitor, induces MDMX degradation and exhibits synergistic cytotoxicity with nutlin-3a *in vitro* and *in vivo* [31]. These results demonstrate that targeting MDMX expression would be of therapeutic benefits. In this study, we demonstrated that MDMX is post-transcriptionally regulated by USP22 in human NSCLC *in vitro* and *in vivo*. USPs, like USP7/HAUSP [32,33] and USP2a [34], were also confirmed to be able to regulate the stabilization of p53 through deubiquitination. However, whether MDMX is stabilized by USP22 through deubiquitylation needs further studies. Measurement of MDMX ubiquitylation in response to USP22 over-expression or down-regulation in NSCLC cells will help confirm this hypothesis. This novel mechanism of MDMX regulation in human NSCLC may potentially be exploited for the development of novel therapies against NSCLC.

USP22 is a cancer-related protein, controls a large variety of genes and, as such, is associated with deregulating signaling altered in cancer [35,36] and suggested as a potential novel target for cancer drugs [9]. An increasing body of evidence has linked USP22 to systemic malignancies. Our study demonstrates that USP22 is highly expressed in NSCLC tissues and plays an important part in NSCLC tumorigenesis, which is consistent with the results obtained from different tumors, such as colorectal carcinoma [11], bladder cancer [13] and prostate adenocarcinoma [37], and contributes to NSCLC tumorigenesis through up-regulating MDMX and subsequently suppressing the p53 pathway. This novel mechanism of MDMX regulation in human NSCLC may potentially be exploited for the development of novel therapies against NSCLC. Furthermore, USP22 silencing presents an inhibition effect on NSCLC tumorigenesis, which makes it an interesting candidate to explore for therapeutic strategies. In the balance, USP22 would be predicted to enhance therapeutic options for the treatment of NSCLC.

## 4. Materials and Methods

### 4.1. Human Tissue Samples and Cell Lines

Normal, paracancerous and NSCLC tissues (*n =* 15) were collected from NSCLC patients enrolled in Xin Hua Hospital affiliated with Shanghai Jiao Tong University School of Medicine. The experimental protocol was approved by the Ethics Committee of Xinhua Hospital and written informed consent was obtained from all patients. Tissue samples were snap-frozen in liquid nitrogen and stored at −80 °C until analysis. The human NSCLC cell lines, A549, SK-MES-1 and NCl-H460, and the noncancerous human bronchial epithelial cell line, 16HBE, were obtained from the Institute of Biochemistry and Cell Biology of the Chinese Academy of Sciences (Shanghai, China). Cells were cultured in RPMI-1640 medium supplemented with 10% fetal bovine serum, 100 IU/mL penicillin and 100 μg/mL streptomycin at 37 °C, 5% CO_2_, in a humidified incubator.

### 4.2. Lentivirus Production and Infection

For the USP22 and MDMX silencing, target-specific shRNAs were designed and synthesized by Shanghai Sangon (Sangon Biotech, Shanghai, China). A scrambled shRNA (SCR) construct was used as a negative control. The target-specific shRNA sequences were as follows: USP22 shRNA, 5'-GAAGCAUUCACGAGCAU-3'; MDMX shRNA, 5'-AAAACUGCCGCUUUUGAAGAU-3'. Then, the lentivirus expressing USP22 shRNA or MDMX shRNA was infected into the specific cell lines. A549 cell line stably expressing Flag-tagged-USP22 or MDMX was also generated by lentiviral infection. Briefly, a lentiviral vector containing the specific shRNA, human USP22 cDNA with an *N*-terminal Flag-tag or human MDMX cDNA sequence, together with psPAX2 and pMD2.G (Tronolab, Geneva, Switzerland), was cotransfected into 293T cells using Lipofectamine 2000 (Invitrogen, Carlsbad, CA, USA). Then, A549 and NCl-H460 cell lines were infected with collected viral supernatants.

### 4.3. Quantitative Real-Time PCR (qRT-PCR)

Total RNA was isolated from frozen tissue samples and cultured cells using TRIzol (Invitrogen) following the manufacturer’s instructions. Reverse transcription was carried out with 2 μg of total RNA from each sample using the SuperScript II RT kit (Invitrogen). Quantitative real-time PCR was conducted using SYBR Green PCR master mix (Applied Biosystems, Foster City, CA, USA) on an ABI 7500HT System (Applied Biosystems). The specific primer sequences for PCR were as follows: USP22, 5'-GACCAGATCTTCACAGGCGG-3' (forward) and 5'-GCAGACTTGGCAGGTGACGT-3' (reverse); MDMX, 5'-GCCTTGAGGAAGGATTGGTA-3' (forward) and 5'-TCGACAATCAGGGACATCAT-3' (reverse); β-actin, 5'-GAGCACAGAGCCTCGCCTTT-3' (forward) and 5'-AGAGGCGTACAGGGATAGCA-3' (reverse). The relative mRNA expression was calculated by the ^ΔΔ^*C*_t_ method.

### 4.4. Immunoprecipitation, Western Blot and Immunofluorescence Analysis

The interaction between USP22 and MDMX in NSCLC cells was studied using co-immunoprecipitation. Briefly, cell lysates were immunoprecipitated with antibodies against Flag, USP22 or MDMX. Precipitated proteins (10 μg) were separated by 12% SDS-PAGE, transferred to nitrocellulose membranes and probed with anti-USP22, anti-MDMX or anti-Flag antibody (Abcam, Cambridge, UK). After incubation with horseradish peroxidase conjugated secondary antibody (Abcam), protein bands were visualized with the ECL kit (Pierce, Rockford, IL, USA). Protein levels in crude cell lysates and tumor tissue homogenates were determined as described above without immunoprecipitation. β-actin was used as a control. For protein expression level analysis, total protein extraction was isolated from frozen tissues or collected cells for western blot analysis, as previously described [17]. Protein bands were visualized as described above. The experiment was repeated at least three times independently. The data reported here are for one representative experiment. For immunofluorescence analysis, A549 cells transfected with USP22 shRNA or control were grown on cover slips, fixed with 3.7% formaldehyde and postfixed with 0.1% Triton X-100 each for 10 min at RT. Photographs were taken 72 h after transfection.

### 4.5. MTT Assay

Transfected A549 and NCI-H460 cells were cultured at 37 °C for 24, 48, 72, 96 and 120 h, respectively. Cells were subsequently incubated with MTT (Sigma–Aldrich, Shanghai, China) at a final concentration of 0.5 mg/mL for 2 h. The reaction was stopped by the addition of 100 μL DMSO, and the optical density at 570 nm was determined on a microplate reader (Bio-Rad, Hercules, CA, USA).

### 4.6. Cell Cycle and Apoptosis Assay

Cell cycle distribution and apoptosis were determined by flow cytometry. Briefly, transfected A549 and NCI-H460 cells were incubated for 48 h, stained with propidium iodide (PI) or double-stained with fluorescein (FITC)-conjugated Annexin V and PI (FITC-Annexin V/PI) and analyzed on a flow cytometer (FACS Calibur, Becton–Dickinson, Franklin Lakes, NJ, USA) to determine cell cycle and rate of apoptosis, respectively.

### 4.7. In Vivo Tumorigenicity

The effect of USP22 silencing on the tumorigenesis of NSCLC *in vivo* was investigated in a mouse xenograft model. A549 cells transfected with SCR shRNA or USP22 shRNA (1 × 10^6^ cells in 100 μL PBS) were injected subcutaneously into the right flank of 5–6-week-old male BALB/c nude mice (Shanghai Experimental Animal Center, Shanghai, China) (*n =* 6). All animal experimental procedures were approved by the Animal Ethics Committee of Shanghai Jiao Tong University. Tumor size was measured once a week over a period of four weeks. The mice were sacrificed, and tumors were harvested and weighed. Protein levels of MDMX and the p53 pathway molecules in tumor tissues were determined by western blot analysis, as described above.

### 4.8. Statistical Analysis

All data analyses were performed using SPSS 13.0 (SPSS Inc., Chicago, IL, USA). Results are expressed as the means ± standard deviation (SD). Differences between two groups were determined by the Student’s *t*-test. Correlation between the protein expressions of USP22 and MDMX or p53 in NSCLC tissues were analyzed using the Pearson correlation coefficient. Differences with *p* < 0.05 were considered statistically significant.

## 5. Conclusions

In summary, our results suggest that USP22 promotes NSCLC tumorigenesis *in vitro* and *in vivo* through MDMX upregulation and subsequent p53 inhibition. USP22 may represent a novel target for NSCLC treatment.
